# Spatially Specialized Nasopharynx–Rectal Microbiomes in Golden Snub-Nosed Monkeys (*Rhinopithecus roxellana*)

**DOI:** 10.3390/biology15171530

**Published:** 2026-09-03

**Authors:** Jie Tang, Nianlong Li, Wenhui Zhou, Ruliang Pan, Lijuan Suo, Baoguo Li, Haitao Zhao

**Affiliations:** 1 Shaanxi Provincial Field Observation & Research Station for Golden Monkey, Giant Panda and Biodiversity, Shaanxi Institute of Zoology, Xi’an 710032, China; yaya184@xab.ac.cn (J.T.);; 2 Shaanxi Key Laboratory for Animal Conservation, Northwest University, Xi’an 710069, China; 3 International Centre of Biodiversity and Primate Conservation, Dali University, Dali 671000, China; 4 School of Human Sciences, University of Western Australia, Perth, WA 6009, Australia; 5 College of Life Science, Yan’an University, Yan’an 716000, China; 6 The School of Technology, Beijing Forestry University, Beijing 100091, China

**Keywords:** golden snub-nosed monkey, nasopharyngeal microbiome, rectal microbiome, functional divergence, co-occurrence network, CAZy, cross-niche interaction

## Abstract

Background: Understanding the nasopharyngeal (NAS) and rectal (INT) microbiomes is critical to maintain host health. However, their functional roles in shaping microbial network structure and niche differentiation remain unexplored in most animal species, especially endangered primates. Here, we compared the NAS and INT microbiomes of golden snub-nosed monkeys (*Rhinopithecus roxellana*) using full-length 16S rRNA amplicon sequencing and metagenomics, focusing on taxonomic composition, functional profiles (KEGG, CAZy, PHI), and microbial co-occurrence networks. Results: Our results indicate that microbial community structure and diversity (Shannon evenness) differ significantly between the two niches. Taxonomically, both sequencing approaches consistently show that, at the relative-abundance level, NAS is dominated by genera such as *Dolosigranulum* and *Vibrio*, whereas the INT is characterized by *Campylobacter*, *Helicobacter*, and *Aerococcus*. Notably, metagenomic analysis indicates a relatively high abundance of potential pathogens, including *Helicobacter pylori* and *Chlamydia psittaci*. LEfSe analysis shows *Dolosigranulum* and *Campylobacter* are key microbial markers for the NAS and INT niches, respectively. KEGG pathway analysis reveals that the NAS microbiome is enriched in valine/leucine/isoleucine degradation and glutathione metabolism, while the INT microbiome is enriched in amino sugar and nucleotide sugar metabolism and ribosome pathways. At the CAZy level, all 29 differentially abundant families (e.g., GH13_29, GH103, AA3_2) were enriched in the NAS. Distinct pathogen–host interaction profiles further reflect niche-specific adaptations. Exploratory Spearman-based co-occurrence networks (in which no genus–CAZy edge survived Benjamini–Hochberg (FDR) correction) linked core commensals to KEGG pathways and CAZy families (e.g., GT35, GH0). In the INT, *Campylobacter* and *Helicobacter* form high-connectivity modules associated with pathways such as those involving the two-component system and ABC transporters. Conclusions: The nasopharyngeal and rectal microbiomes in golden snub-nosed monkeys differ not only taxonomically but also functionally, exhibiting clear spatially specialized functional differentiation. Given the small sample size (n = 8), these findings provide preliminary evidence for niche-specific microbial and functional partitioning and suggest that the nasopharynx may harbor a relatively high abundance of potential zoonotic pathogens. This study provides a basis for further exploration with a larger sample size.

## Simple Summary

The golden snub-nosed monkey is an endangered primate, and understanding the microbes that live in different parts of its body could help us protect both the species and the broader ecosystems it inhabits. In this study, we compared the microbial communities living in the nasopharynx and rectum of these monkeys using full-length 16S rRNA amplicon sequencing and metagenomic analysis. We found that the two body sites host clearly different microbial communities. Functionally, the nasopharynx microbiome was enriched in pathways for breaking down certain amino acids and for antioxidant (glutathione) defense, while the gut microbiome specialized in processing sugars and building proteins. Notably, we detected relatively high levels of potentially disease-causing bacteria in the nasopharynx, including *Helicobacter pylori* and *Chlamydia psittaci*. These findings suggest that the nasopharynx and rectum represent distinct microbial environments in this endangered primate and highlight the nasopharynx as a possible reservoir of zoonotic pathogens, although larger studies are needed to confirm these patterns.

## 1. Background

The animal microbiome, the “second genome” of host organisms [1], exhibits remarkable complexity and dynamic properties that profoundly influence host nutritional metabolism [2], immune defense [3], and ecological adaptation strategies [4]. Recent breakthroughs in microbiome research have underscored its indispensable role in preserving host homeostasis and mediating environmental acclimatization, and the field has attracted great attention as a frontier of life sciences [5,6]. In particular, cross-niche microbial interactions—via metabolite signaling, immune cell trafficking, or behavioral transmission—are increasingly recognized as a key factor in understanding host health and evolutionary adaptation.

The golden snub-nosed monkey (*Rhinopithecus roxellana*), as a typical arboreal primate species, has a unique ecological niche and diet, and a unique multi-level selective pressure on its microbiota [7,8]. In humans and nonhuman primates, resident microorganisms form in response to environmental factors, social interactions, and cohabitation [9,10]. Moeller et al. [11] have demonstrated that the frequency of social contact is strongly associated with the exchange of microorganisms between hosts. This exchange can occur either through direct physical contact or indirectly via environmental transmission.

Currently, research on the microbiome of the golden snub-nosed monkey focuses mainly on a single ecological niche (the intestine), including analyses of microbial composition [12,13,14] and the influence of seasonally driven diets on microbiome plasticity [7], and evidence that dietary and nutrient composition drive the structural and functional reorganization of gastrointestinal microorganisms [15]. However, the compositional characteristics of microbial communities in other body sites and the potential functional complementarity among ecological niches have been overlooked.

Both the nasopharynx and the gastrointestinal tract are mucosal immune compartments that share fundamental architectural and immunological features, including a polarized epithelial barrier, a mucus layer, and mucosa-associated lymphoid tissues [16,17]. The nasopharynx, the interface between the respiratory tract and the outside body, is the first line of defense against pathogens; it can thus be viewed as a gatekeeper of respiratory health [18]. The gastrointestinal tract is the core site for nutrient absorption and microbial fermentation [19]. At both mucosal sites, epithelial cells actively participate in microbiome assembly through the secretion of antimicrobial peptides—such as defensins and regenerating islet-derived protein 3γ (REG3γ)—and mucus glycoproteins that selectively constrain microbial colonization to the luminal niche [20,21]. In addition, secretory immunoglobulin A (sIgA), the predominant antibody isotype at mucosal surfaces, coats commensal bacteria and modulates their spatial organization and community composition [22]. Importantly, these locally acting mechanisms are integrated within the common mucosal immune system, in which lymphocytes primed at one mucosal inductive site can recirculate and home to distant mucosal effector sites, thereby functionally linking immune surveillance across the respiratory and gastrointestinal compartments [17]. These shared and interconnected mucosal immune pathways suggest that the microbial communities of the nasopharynx and the gut may interact not only through metabolic product cycling but also through immune-mediated cross-talk, making their cross-niche interaction network a potentially important yet underexplored dimension of host adaptation.

This study aims to integrate full-length 16S rRNA gene and metagenomic sequencing data to systematically characterize the microbial communities of the NAS and INT in a free-ranging *Rhinopithecus roxellana*. We address the following issues: (i) Spatial distribution specificity: What are the compositional characteristics of the NAS microbiota in the monkeys, and how do they differ from or resemble those of the rectal microbiota? (ii) Cross-niche microbial interactions: Do the NAS and INT microbiotas form specialized interaction networks? Which taxa act as keystone species mediating cross-niche microbial associations? (iii) Functional relevance to host adaptation: how do spatially specialized microbiome configurations relate to host adaptive strategies?

## 2. Materials and Methods

### 2.1. Sample Collection

The experimental samples were collected from the Guanyinshan National Nature Reserve, Qinling Mountains, China (107°52′–108°02′ E, 33°20′–33°44′ N). Swabs from the nasopharynx and fecal samples were collected from 8 monkeys from July 2024 to May 2026. All monkeys were sourced from progeny supplementation and the field from the Rescue, Breeding and Research Center. Based on physical characteristics and dental structure, they were estimated to be between 5 and 12 years old: two sub-adult males (approximately 5–6 years old), two adult males (approximately 10–12 years old), and four adult females (approximately 6–9 years old). The two sub-adult males came from an all-male unit (bachelor group). The four females and the two adult males belonged to different family groups (one female and one male per family group). All individuals appeared healthy at the time of capture, with no visible signs of injury, nasopharynx discharge, diarrhea, or other clinical abnormalities.

All monkeys were captured by professional technicians under veterinary supervision from the Shaanxi Rare Wildlife Rescue, Breeding and Research Center, using remote drug-delivery dart guns (blowpipe). Anesthesia was induced with Zoletil^®^ 50 (Virbac, Carros, France) (tiletamine-zolazepam) at a dose of 4 mg/kg. During anesthesia (duration approximately 30–40 min), physiological monitoring was conducted every 10 min, including heart rate (stethoscope) and respiratory rate measurements. Body temperature was maintained using a warm blanket. Upon full recovery, each individual was released at the exact site of capture. Post-release monitoring via tracking or visual observation confirmed that all individuals returned to their groups and behaved normally within 2 h. No adverse events, such as hypothermia, bradycardia, apnea, or injury, occurred during or after the procedure.

For rectal swab collection, an aseptic swab was inserted about 4 cm into the anus and rotated 3 times to ensure full contact with the rectal mucosa. As for nasopharynx swab collection, a sterile swab was carefully inserted into the nostril and gently advanced along the floor of the nasal cavity toward the nasopharynx until resistance was felt. The swab was then rotated gently to collect epithelial cells and secretions before being slowly withdrawn. All swabs collected were immediately transferred to sterile cryotubes and stored in liquid nitrogen for subsequent 16S full-length sequencing and metagenomic sequencing.

Given the limited sample size from this endangered species, each sample was processed as a single biological replicate, and no technical replicates were conducted.

### 2.2. Full-Length 16S rRNA Gene Sequencing

There were 16 genomic DNA samples in total (from the nasopharynx and rectal of each sampled monkey), collected using the TGuide S96 Magnetic Soil/Stool DNA Kit (Tiangen Biotech (Beijing) Co., Ltd., Beijing, China). The V1-V9 hypervariable regions of the 16S rRNA gene were amplified with primers (27F: AGRGTTTGATYNTGGCTCAG; 1492R: TASGGHTACCTTGTTASGACTT). The amplicons were quantified, and the normalized equimolar concentrations were pooled and sequenced on the PacBio Sequel II platform (Beijing Biomarker Technologies Co., Ltd., Beijing, China).

### 2.3. Metagenome Sequencing

According to [23], the following procedures were performed: the raw reads were quality-filtered using Fastp (v 0.23.1). Host contamination was removed by aligning reads against the host genome (Genome assembly ASM756505v1) using bowtie2 (v2.2.4), yielding clean reads. The clean reads were assembled with MEGAHIT (v1.2.1) using default parameters, and contigs shorter than 300 bp were discarded.

Coding genes were predicted from the assembled contigs using MetaGeneMark software (http://exon.gatech.edu/meta_gmhmmp.cgi, accessed on 15 May 2026, Version 3.26), with default parameters. MMseqs2 (https://github.com/soedinglab/mmseqs2, accessed on 20 May 2026, Version 17-b804f) was used to remove redundancy with a similarity threshold set to 95%, and the coverage threshold to 90%.

Taxonomic profiling of the metagenomic data was performed as follows. The predicted protein sequences were aligned against the Non-Redundant Protein Sequence Database (NR) using diamond (v0.9.29) with the following parameters: E-value ≤ 1 × 10^−5^, sequence identity ≥ 60% (--id 60), and query coverage ≥ 15% (--query-cover 15). For each gene, the best-scoring single match was retained (best-hit approach). Taxonomic assignments were made at each level (phylum, class, order, family, genus, species) based on the best-hit matches. Gene abundance quantification was performed by mapping clean reads to the non-redundant gene catalog using BWA (v0.7.10). Mapped reads were counted using samtools (v1.9) to obtain absolute abundance (reads per gene). Taxonomic abundance at each level (domain to species) was calculated by aggregating gene-level absolute abundance of all genes assigned to each taxon. Relative abundance was then calculated as each taxon’s summed absolute abundance divided by the total abundance of all taxa at that level within the same sample.

To obtain gene functional annotations, the protein sequences of the predicted genes were compared with functional databases using BLAST-based methods (diamond v0.9.29, E-value 1 × 10^−5^), including the Non-Redundant Protein Sequence Database (NR), Kyoto Encyclopedia of Genes and Genomes (KEGG), Gene Ontology (GO), and Pathogen Host Interactions Database (PHI-base) (version 4.6.1). The annotated gene abundance tables were compared with the GO and KEGG databases to obtain protein, functional, and metabolic pathway information. HMMER (v3.0) software was used to compare protein sequences of non-redundant genes with the Hidden Markov models of each family in the CAZy database, identifying all families that meet the filtering threshold, and carbohydrate-active enzymes in the genome.

### 2.4. Bioinformatics Analysis

The UCHIME algorithm (v8.1) was used to detect and remove chimera sequences to obtain the clean reads. Sequences with more than 97% similarity thresholds were assigned to one operational taxonomic unit (OTU) using USEARCH (v 10.0) [24], and OTUs with abundance <0.005% were filtered out. Taxonomy annotation of the OTUs was performed using the Naive Bayes classifier in QIIME2 (version 2020.6) [25] with the SILVA database [26] (release 138.1) and a confidence threshold of 70%. Meanwhile, Alpha diversity (Chao1 and Shannon) was calculated and displayed using QIIME2 and R (v3.5.2). Differences between paired groups were assessed by paired Wilcoxon signed-rank tests to account for the paired design. To examine Beta diversity (among samples), principal coordinates analysis (PCoA) and Non-Metric Multidimensional Scaling (NMDS) were performed based on binary Jaccard distances at the OTU level using QIIME2. Community dissimilarity between groups under the paired design was assessed using permutational multivariate analysis of variance (PERMANOVA) with permutation constraints at the individual block level.

To further compare bacterial communities across samples, PERMANOVA was performed using the vegan package (2.3-0) in R. Linear discriminant analysis (LDA) was coupled with effect size (LEfSe), identifying representative species. LDA was conducted from the genus to species level [27]. LDA scores ≥ 4.0 and *p* < 0.05 were considered signature taxa and selected for plotting and further analysis.

Differential abundance of the GO level-2 function, KEGG level-3 pathways, CAZy, and PHI annotations was assessed. For each comparison, the Wilcoxon rank-sum test was performed, and the results were corrected for multiple testing using the Benjamini–Hochberg FDR method. To explore potential microbial co-occurrence patterns within each niche, SparCC correlation analysis (1000 permutations) was performed among the top 30 most abundant genera in each group; co-occurrence networks were visualized using edges with |r| > 0.6 for descriptive purposes. Spearman’s rank correlation analysis was conducted to construct bipartite correlation networks between the top 30 genera and CAZy families/KEGG functions, and correlations with |r| ≥ 0.6 and adjusted *p* < 0.05 were considered statistically significant. All analyses were conducted on the BMKCloud platform (www.biocloud.net).

## 3. Results

### 3.1. Sequencing Quality and Microbial Diversity in the NAS and INT Microbiomes

Following full-length 16S rRNA amplification and sequencing, 966,597 CCS sequences of 16 swab samples were obtained. Each yielded an average of 60,412 CCS sequences (Supplementary Table S1). The dilution curve is stable, as shown in Figure S1 in the Supplementary Materials, indicating that the sequencing depth has adequately covered the majority of operational taxonomic units (OTUs), thereby verifying the completeness of microbial diversity capture and the reliability of the results. After quality control of the sequencing data, a total of 143.53 Gb of clean data was obtained. The number of reads per sample ranged from 42,538,218,210 to 82,724,588, with an average of 58,957,953 bp, and the Q30 was 94.73%. An average of 73,540 assembly contigs per sample was identified, yielding an average total assembly length of 66,239,031 bp per sample and an average contig length of approximately 900.71 bp (Supplementary Table S2). The majority of contigs ranged from 500 to 1000 bp (Supplementary Figure S2). The sequencing data quality is reliable for subsequent analysis.

**Figure 1:**
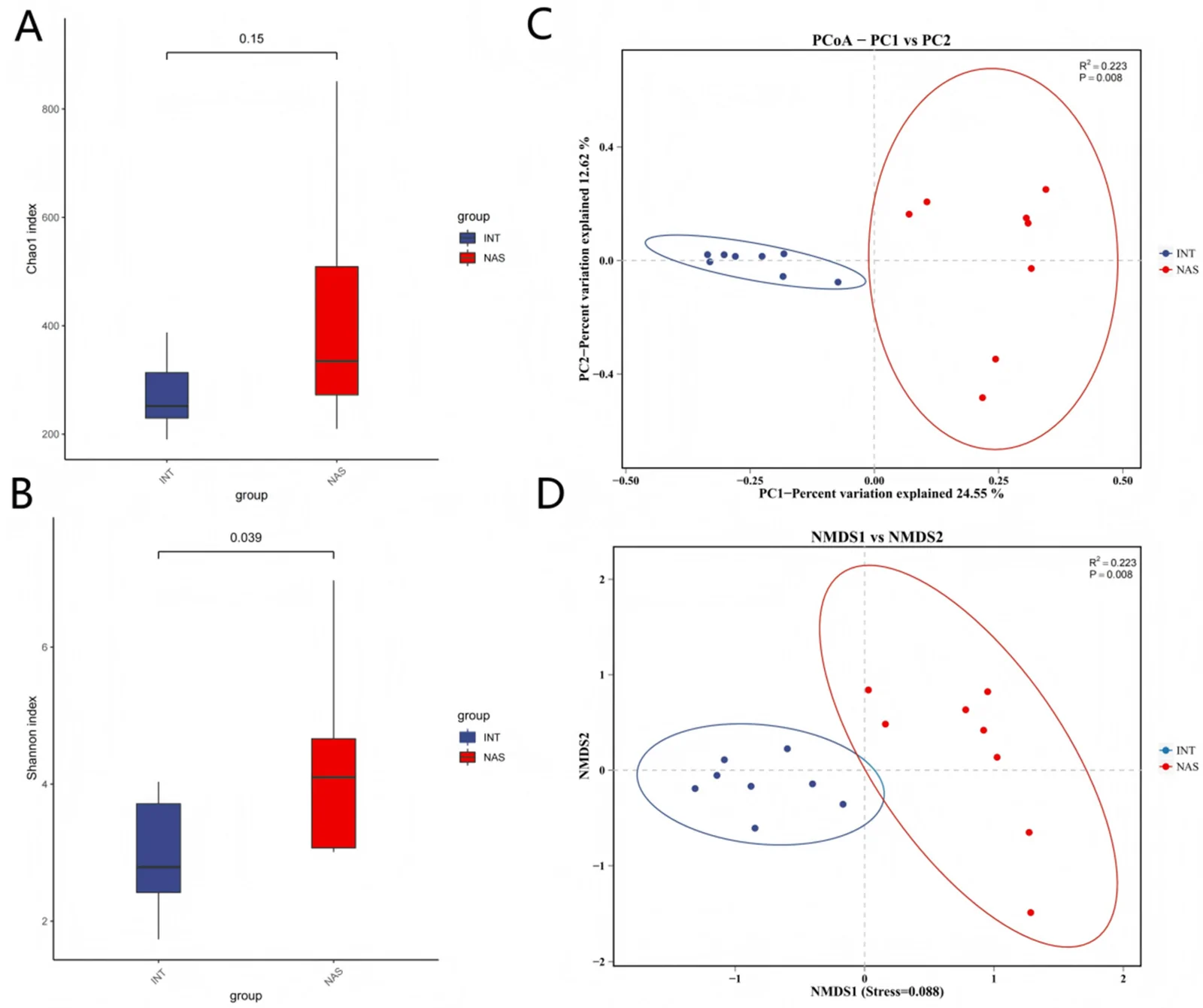
Diversity profiles of the NAS and INT groups based on full-length 16S rRNA gene sequencing. (**A**) Chao1 index. (**B**) Shannon index. (**C**) PCoA based on binary Jaccard distances. (**D**) NMDS analysis (Stress = 0.088).

Further analysis of microbial diversity between the NAS and INT sample groups revealed that the Chao1 index, which primarily reflects the species richness of the microbial community, is slightly higher in the NAS group, although this difference was not significant (Figure 1A; *p* = 0.15). In contrast, the Shannon index, which mainly reflects the community evenness of the microbiota, was significantly higher in the NAS group, indicating a more evenly distributed diverse microbial community (Figure 1B; *p* = 0.039). For beta diversity, PCoA based on the binary Jaccard distances revealed a clear separation between the NAS and INT groups along PC1 (Figure 1C; PERMANOVA R^2^ = 0.223, *p* = 0.008). The INT samples formed a tight cluster, whereas the NAS samples exhibited greater within-group dispersion. A complementary NMDS analysis (Stress = 0.088) showed a consistent pattern of group separation (Figure 1D).

### 3.2. Microbial Community Structure and LEfSe Analysis

**Figure 2:**
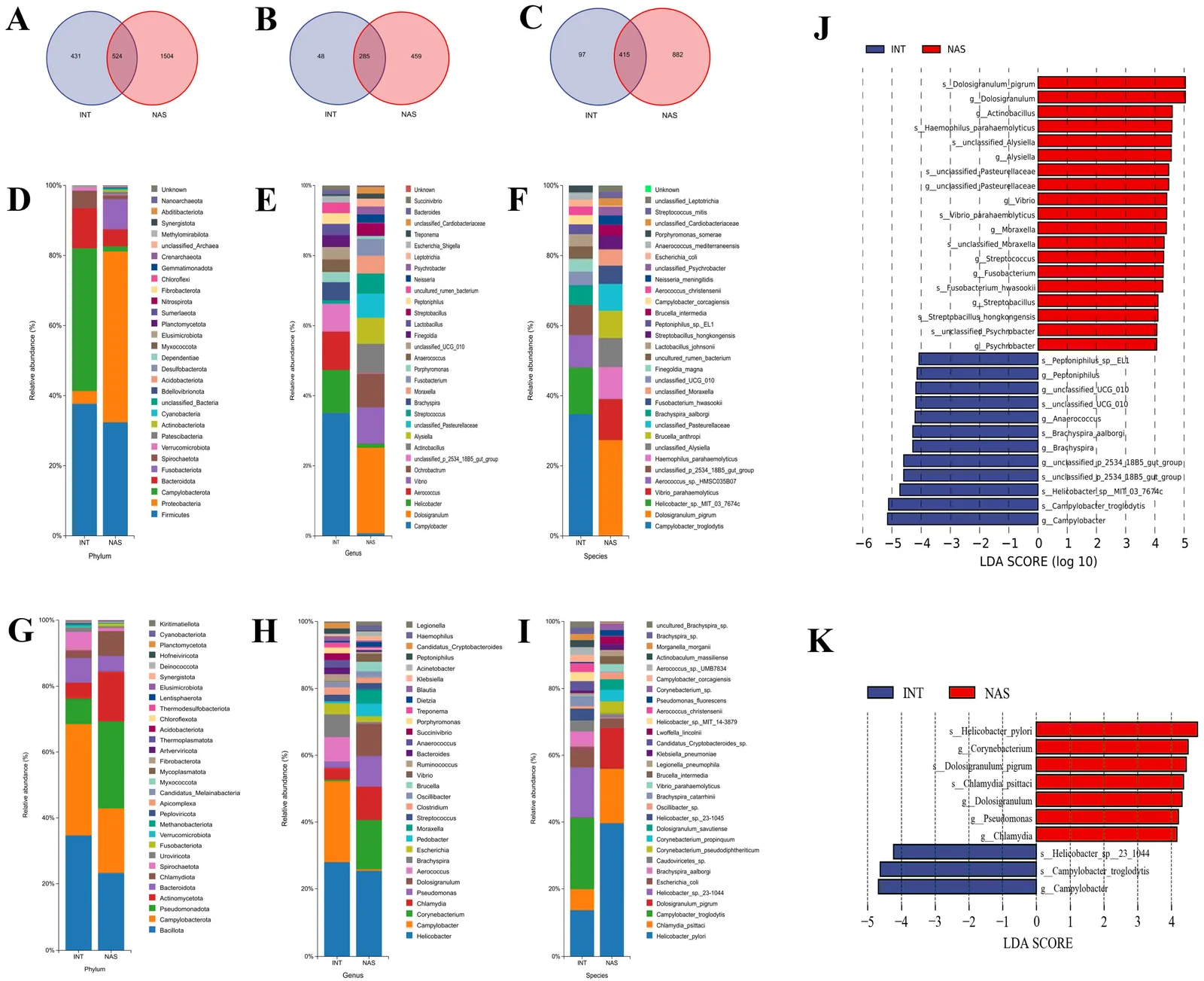
Microbiome diversity comparison between NAS and INT in golden snub-nosed monkeys, explored with full-length 16S and metagenomic sequencing. (**A**–**C**): A Venn diagram illustrates the numbers of unique and shared OTUs (**A**), genus (**B**), and species (**C**) between the NAS and INT microbiomes. (**D**–**F**): Stacked bar charts display relative abundance at phylum (**D**), genus (**E**), and species (**F**) levels, based on 16S full-length sequencing. (**G**–**I**): Stacked bar charts show microbial relative abundance at the phylum (**G**), genus (**H**), and species (**I**) levels, based on metagenomic sequencing. (**J**): LEfSe analysis of full-length 16S rRNA gene sequencing that identifies microbial biomarkers (LDA score > 4.0), distinguishing NAS (red) and INT (blue) microbiota; (**K**): and LEfSe analysis of metagenomic sequencing data that confirm niche-specific microbial signatures (LDA score ≥ 4.0). Note: *Y*-axis: Features that show a difference between groups; *X*-axis: Log10 of LDA score, where the LDA score threshold is 4.0. The features are sorted by LDA score. The longer the bar is, the more significant the difference level becomes.

Full-length 16S rRNA sequencing revealed niche differentiation between NAS and INT microbiota in this small cohort. Among 2459 OTUs (97% similarity), NAS contained more OTUs (2028) than INT (955), with 524 shared between the two niches (Figure 2A). A core microbiota of 285 genera and 415 species coexisted with niche-specific assemblages, reflecting both conservation and adaptation across anatomical sites (Figure 2B,C).

Taxonomic profiles by 16S full-length sequencing (Figure 2D–F) and metagenomic sequencing (Figure 2G–I) show distinct microbial community structures between NAS and INT at the phylum, genus, and species levels. At the phylum level (Figure 2D,G), INT communities were dominated by Campylobacterota and Firmicutes in both datasets, whereas NAS communities were dominated by Proteobacteria and Firmicutes. At the genus level (Figure 2E,H), 16S sequencing revealed distinct dominant taxa between the two niches: the NAS group was characterized by *Dolosigranulum* and *Vibrio* (Figure 2E); the INT group was dominated by *Campylobacter*, *Helicobacter*, and *Aerococcus*, (Figure 2E). Of the top 30 most abundant genera, 14 were shared by the two sequencing approaches (16S and metagenomics) (Supplementary Figure S3): *Dolosigranulum*, *Helicobacter*, and *Vibrio* in NAS, and *Helicobacter*, *Campylobacter*, and *Aerococcus* in INT. The two sequencing approaches exhibited complementary detection scopes: 16S sequencing uniquely identified 16 genera, primarily comprising cultivable or common bacteria, while metagenomics uniquely classified 16 genera, including taxa undetected by 16S (such as *Corynebacterium*, *Chlamydia*, and *Pseudomonas*) (Supplementary Table S3).

At the species level, the two methods were largely concordant—*Campylobacter troglodytis* was the predominant species in INT in both datasets, and *Dolosigranulum pigrum* was the leading species in NAS by 16S (19.43%). Metagenomics further resolved *Helicobacter pylori* as the most abundant species in NAS (19.26%) and detected potential zoonotic pathogens, most notably *Chlamydia psittaci* (7.88%), which 16S did not recover at a comparable abundance. Overall, some species were recovered by both approaches (including *Campylobacter troglodytis*, *Dolosigranulum pigrum*, and *Escherichia coli*), suggesting the consistency of the two sequencing approaches (16S and metagenomics) at the species level while highlighting metagenomics’ added resolution for fastidious taxa (Figure 2F,I).

The LEfSe analysis (LDA > 4.0) identified distinct microbial biomarkers for each niche. In 16S rRNA data (Figure 2J), NAS microbiota was enriched in *Dolosigranulum* (*D. pigrum*), *Actinobacillus*, *Haemophilus parahaemolyticus*, *Vibrio*, *Streptobacillus* (*S. hongkongensis*), *Fusobacterium* (*F. hwasookii*), and *Psychrobacter*. In contrast, INT microbiota was dominated by gut-adapted taxa, including *Peptoniphilus*, *Anaerococcus*, *Brachyspira aalborgi*, and *Campylobacter* (*C. troglodytis*). Metagenomic analysis (Figure 2K) further identified NAS-specific biomarkers not captured by 16S sequencing, including *Helicobacter pylori*, *Corynebacterium*, *Pseudomonas*, and *Chlamydia*. Both sequencing platforms identified *Campylobacter* as rectal biomarkers.

### 3.3. Within-Niche Microbial Co-Occurrence Networks

To preliminarily explore potential microbial co-occurrence patterns within each niche, SparCC correlation networks were constructed among the top 30 most abundant genera for each groups. Given the limited sample size (n = 8 per niche), the resulting networks are presented as exploratory and require experimental validation. Because correlations with |r| ≥ 0.9 are prone to be numerical artifacts of compositional structure and small sample size, at this sample size, all such edges (9 in the NAS network and 13 in the INT network; full list in Supplementary Table S4) were flagged as pseudo-correlations, shown as gray-dotted lines in the figures, and excluded from biological interpretation. After Benjamini–Hochberg correction, no correlation remained significant at q < 0.05. Our interpretation therefore focuses on moderate correlations (0.6 < |r| < 0.9) and global modular structures. These patterns are exploratory and do not imply cross-niche microbial interactions; they require validation in a larger cohort.

**Figure 3:**
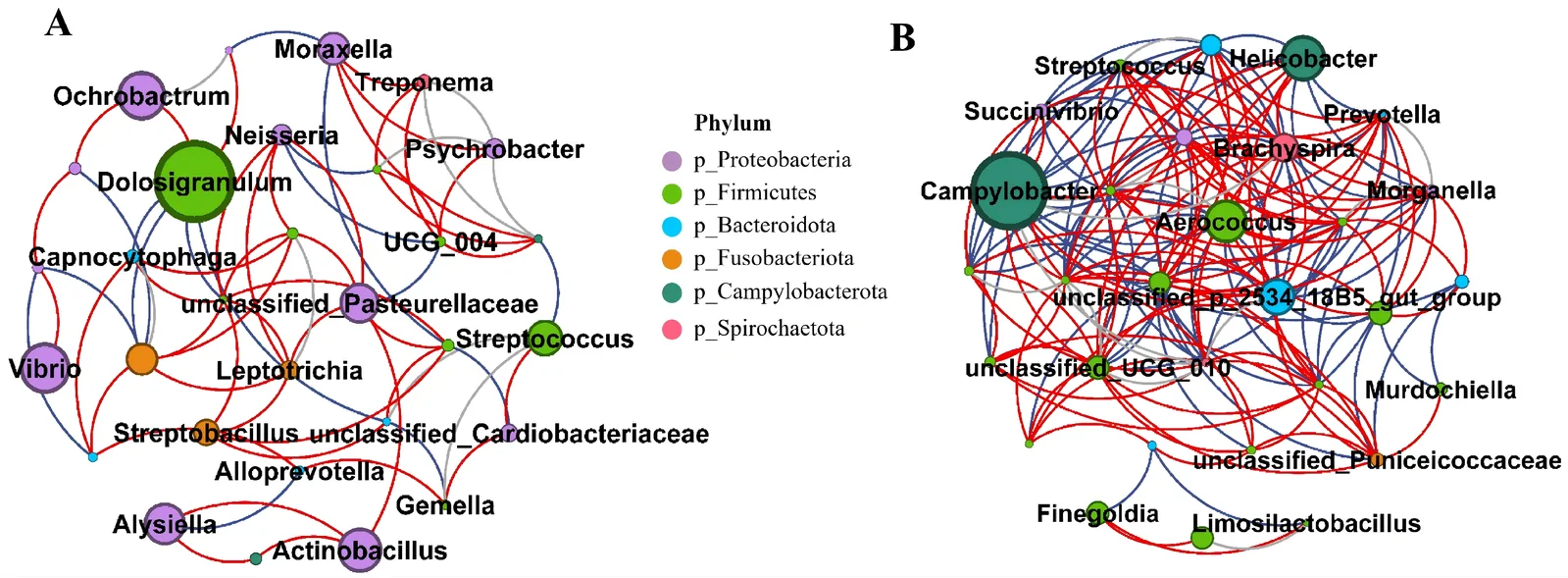
Exploratory genus-level microbial co-occurrence networks in NAS and INT microbiomes. Networks were constructed from SparCC correlations (1000 permutations) based on full-length 16S rRNA gene sequencing among the top 30 abundant genera in each group (n = 8 samples per group; edges shown for |r| > 0.6). (**A**): The correlation network diagram of the NAS group. (**B**): The correlation network diagram of the INT group. Red lines represent positive correlations with r < 0.9, blue lines show negative correlations with r < −0.9, gray lines show a relationship with |r| > 0.9; the color of nodes means different phyla of core bacterial genera, and the size of nodes indicates the abundance of each genus.

In the NAS network (Figure 3A, Supplementary Table S4-1), *Johnsonella* and *Fusobacterium* showed the highest connectivity (degree = 8), followed by *Dolosigranulum*, *Veillonella*, and *Neisseria*. *Dolosigranulum* was negatively associated with *Capnocytophaga* (r = −0.69), *Bergeyella* (r = −0.65), and *Johnsonella* (r = −0.65), and positively associated with *Ochrobactrum* (r = 0.70) and *Aeromonas* (r = 0.65). *Johnsonella* was positively associated with *Veillonella* (r = 0.88) and *Capnocytophaga* (r = 0.70). As for the INT network (Figure 3B, Supplementary Table S4-2), *Treponema* formed a tightly associated module, positively correlated with *Ruminococcus* (r = 0.88) and *Brachyspira* (r = 0.71). Negative correlations were observed between *unclassified_Puniceicoccaceae* and *Anaerococcus* (r = −0.87), as well as between *Murdochiella* and *Bacteroides* (r = −0.89).

Comparisons of the two independent within-niche networks identified five genera in both niches, including *Campylobacter*, *Treponema*, *Porphyromonas*, *Helicobacter*, and *Streptococcus*. *Porphyromonas* displayed niche-dependent association patterns: it negatively correlated with *Stenotrophomonas* (r = −0.79) in the NAS and with *Brachyspira* in the INT (r = −0.74), whereas it showed positive associations with *Escherichia-Shigella* (r = 0.89) in the INT group.

### 3.4. Functional Profiling and Cross-Niche Correlation of the Microbiome

To further characterize functional divergence between the NAS and INT microbiomes, functional profiles were inferred using multiple bioinformatic pipelines. The two niches exhibited distinct, pathway-specific functional configurations.

**Figure 4:**
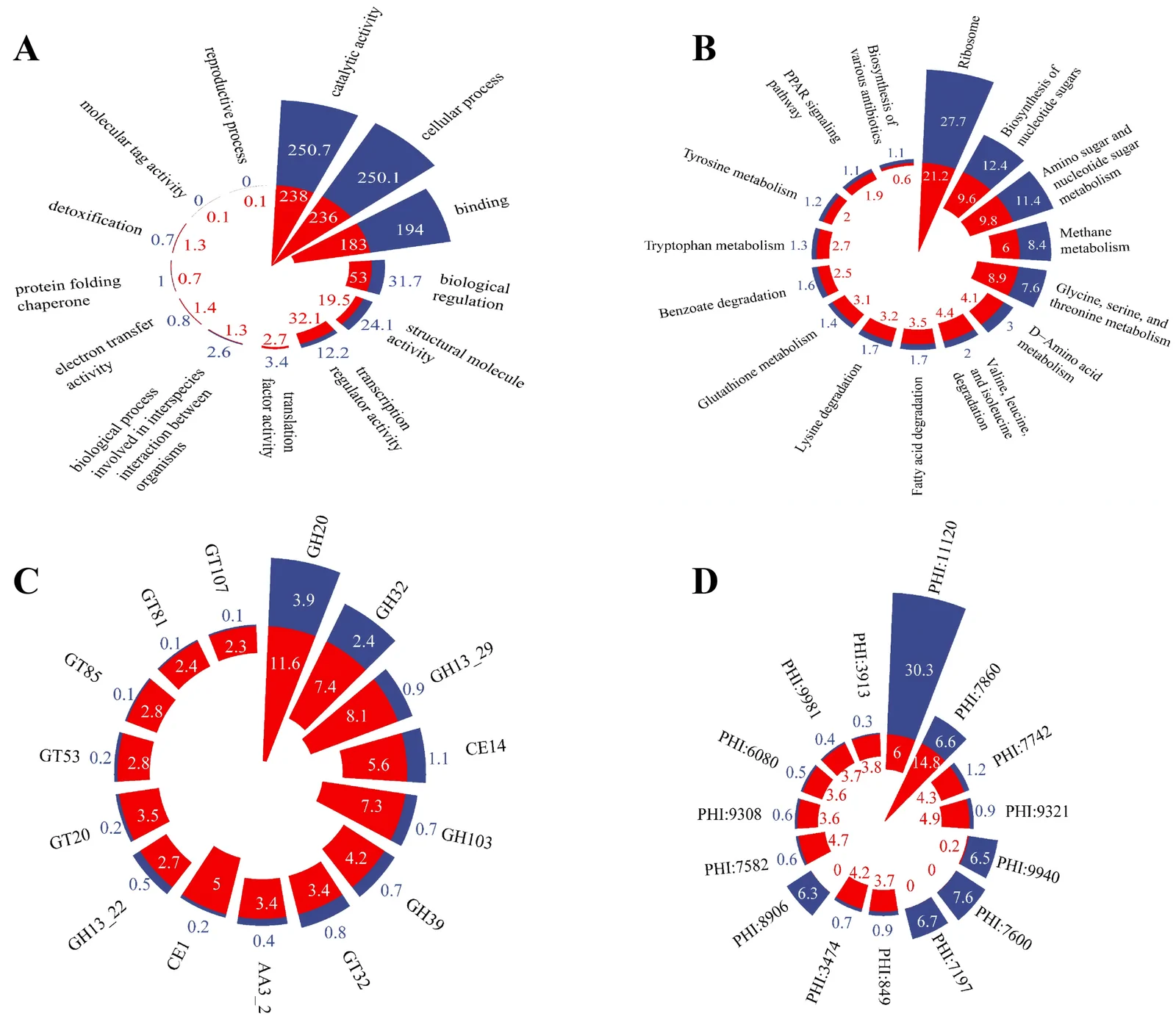
Functional profiles of the NAS and INT microbiota in golden snub-nosed monkeys based on metagenomic sequencing. (**A**): Relative abundance of Gene Ontology (GO) functional categories at level 2. (**B**): Differences in abundance of KEGG-Level 3 pathways between NAS and INT. (**C**): Abundance comparison of CAZyme families between the NAS and INT groups. (**D**): Differential abundance of pathogen–host interaction (PHI) functions between the NAS and INT groups. Statistical significance is determined by the Wilcoxon rank-sum test with BH-FDR correction. Blue represents INT, and red indicates the NAS group. The top 15 abundance rankings are sorted in descending order by adjusted *p*-values and then by the maximum absolute differences in mean relative abundance between NA and INT microbiomes.

Gene Ontology (GO) analysis at the second level identified 13 functional categories that were significantly differentiated between the two microbiomes (adjusted *p* < 0.05). Of these, seven were enriched in the INT microbiome—including cellular process, catalytic activity, binding, structural molecule activity, protein folding chaperone, translation factor activity, and interspecies-interaction processes—whereas six were enriched in the NAS microbiome, notably, biological regulation, transcription regulator activity, detoxification, reproductive process, electron transfer activity, and molecular tag activity (Figure 4A). The KEGG level-3 pathway revealed 62 significantly differential pathways (adjusted *p* < 0.05). The INT-enriched pathways included ribosome, amino sugar, and nucleotide sugar metabolism; methane metabolism; and biosynthesis of various antibiotics. In contrast, the NAS microbiome was predominantly characterized by valine, leucine, and isoleucine degradation; glutathione metabolism; and fatty acid degradation (Figure 4B, Supplementary Table S5). At the CAZy family level (Figure 4C, Supplementary Table S6), 29 families showed differences (adjusted *p* < 0.05), and all were enriched in the NAS microbiome (e.g., GH13_29, GH103, CE14, GH39, AA3_2). Analyzed at the CAZy class level, the Auxiliary Activities (AA) class was significantly enriched in the NAS microbiome (mean relative abundance 0.94% ± 0.07% vs. 0.27% ± 0.04%; adjusted *p* = 0.0047). Pathogen–host interaction (PHI) functional profiles revealed site-specific enrichment patterns. Among the 134 quantified PHI categories, all showed highly significant differences in mean relative abundance between the two niches (adjusted *p* < 0.05), with the majority (126/134) enriched in the NAS microbiome and only eight in the INT microbiome. The INT microbiome was significantly enriched for PHI functions (e.g., PHI:11120, PHI:7600, and PHI:7197. In contrast, the NAS microbiome showed higher abundance of PHI functions (e.g., PHI:7860, PHI:7582, and PHI:3474). Notably, functions like PHI:7197 were nearly exclusive to the INT niche, with negligible detection in NAS, whereas PHI:4573, PHI:5575, PHI:7218, PHI:4667, and PHI:11189 were retained in the NAS microbiome (Figure 4D, Supplementary Table S7).

### 3.5. Correlation Network Analysis of Nasopharynx and Rectal Microbiota with KEGG Pathways and CAZy

**Figure 5:**
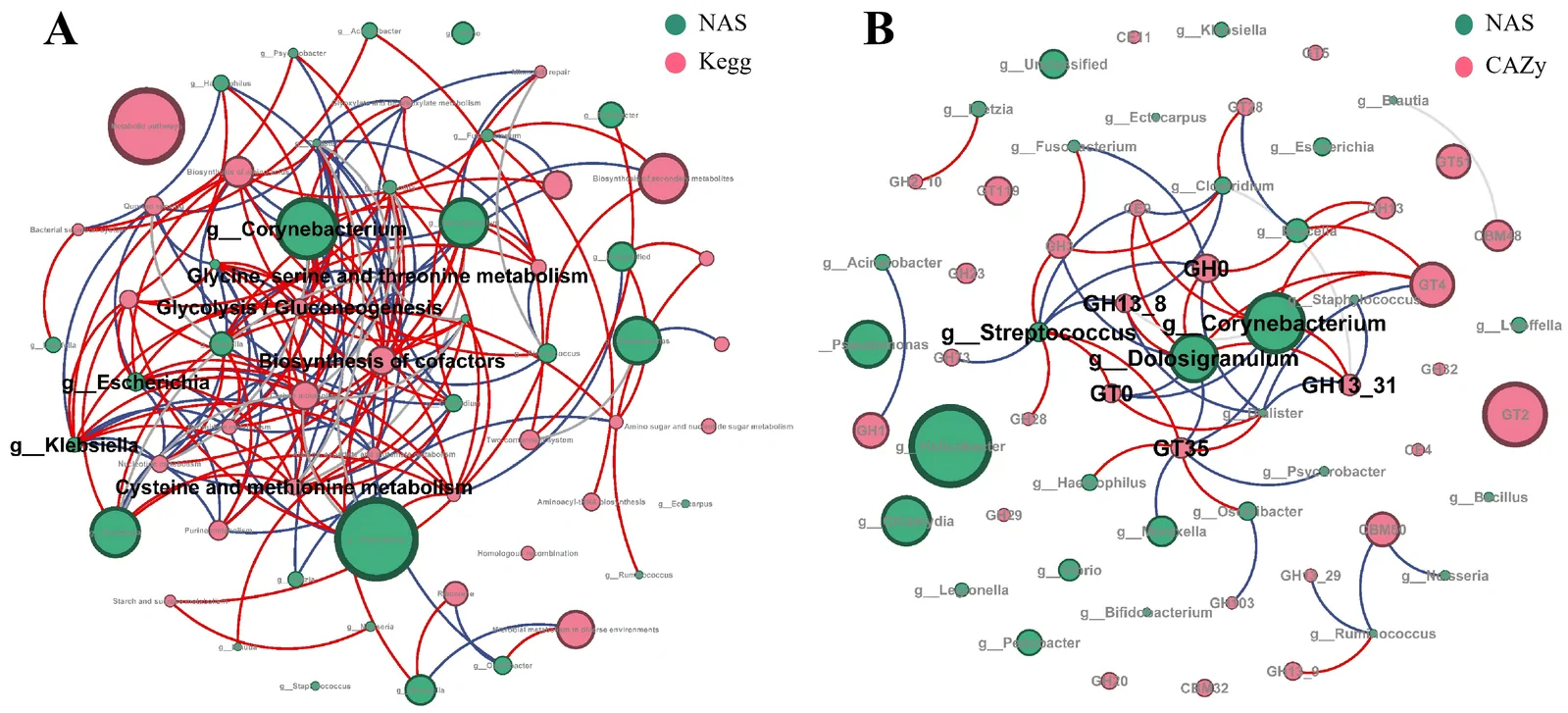
Microbial-KEGG/CAZy co-occurrence networks in the NAS microbiomes. (**A**): Co-occurrence network of core microbes and KEGG pathways in the top 30 NAS microbiome. (**B**): Co-occurrence network of core microbes and CAZy families in the top 30 NAS microbiome. Red lines show a positive relationship with r < 0.9, while blue lines show a negative relationship with r > −0.9. Gray lines show a relationship with |r| > 0.9.

Co-occurrence networks between the top 30 genera and CAZy families/KEGG functions were constructed using Spearman’s correlation. Candidate edges were first screened at |r| ≥ 0.6 and p-raw < 0.05, followed by BH–FDR correction (qBH < 0.05). In the genus–CAZy networks, no edge remained significant after correction (minimum qBH = 0.078). In the genus–KEGG networks, 19 edges met the significance threshold; however, all of these exhibited |r| ≥ 0.9 (Figure 5, Supplementary Tables S8 and S9). Given the limited sample size (n = 8 per anatomical site), such extreme correlations are the classic signature of numerical or compositional artifacts and are therefore set aside as unstable in the following analysis. All substantive interpretations below are restricted to exploratory associations within the 0.6 ≤ |r| < 0.9 range (all q_BH > 0.05). Complete edge-level data for all networks are provided in Supplementary Tables S8-1–S9-2.

In the NAS genus–KEGG network (Figure 5A), the most extensively connected pathways were biosynthesis of cofactors (12 genus links), glycolysis/gluconeogenesis (12), pyrimidine metabolism (11), and biosynthesis of amino acids (10). Biosynthesis of cofactors showed a consistent negative association with most genera (e.g., *Dialister* r = −0.88, *Helicobacter* r = −0.71, *Chlamydia* r = −0.71, *Haemophilus* r = −0.76, *Klebsiella* r = −0.69, *Bacillus* r = −0.69) but positive links with *Corynebacterium* (r = +0.86), *Dolosigranulum* (r = +0.83), and *Moraxella* (r = +0.81). Glycolysis/gluconeogenesis was negatively associated with *Dolosigranulum* (r = −0.83) yet positively with *Helicobacter* (r = +0.81). Biosynthesis of amino acids showed an opposing pattern for *Dolosigranulum* (r = −0.88) versus Streptococcus (r = +0.86). Glycine, serine, and threonine metabolism appeared via links (e.g., *Dolosigranulum* r = −0.81, *Corynebacterium* r = −0.76, *Clostridium* r = +0.71). Across these pathways, *Corynebacterium* and *Dolosigranulum* emerged as recurring hubs, with particularly strong positive links to multiple pathways including alanine, aspartate, and glutamate metabolism. In the NAS genus–CAZy network (Figure 5B), no edge remained significant after Benjamini–Hochberg (FDR) correction; the most connected were *Dolosigranulum* and *Streptococcus* (eight CAZy-family links each) and *Corynebacterium* (eight links).

**Figure 6:**
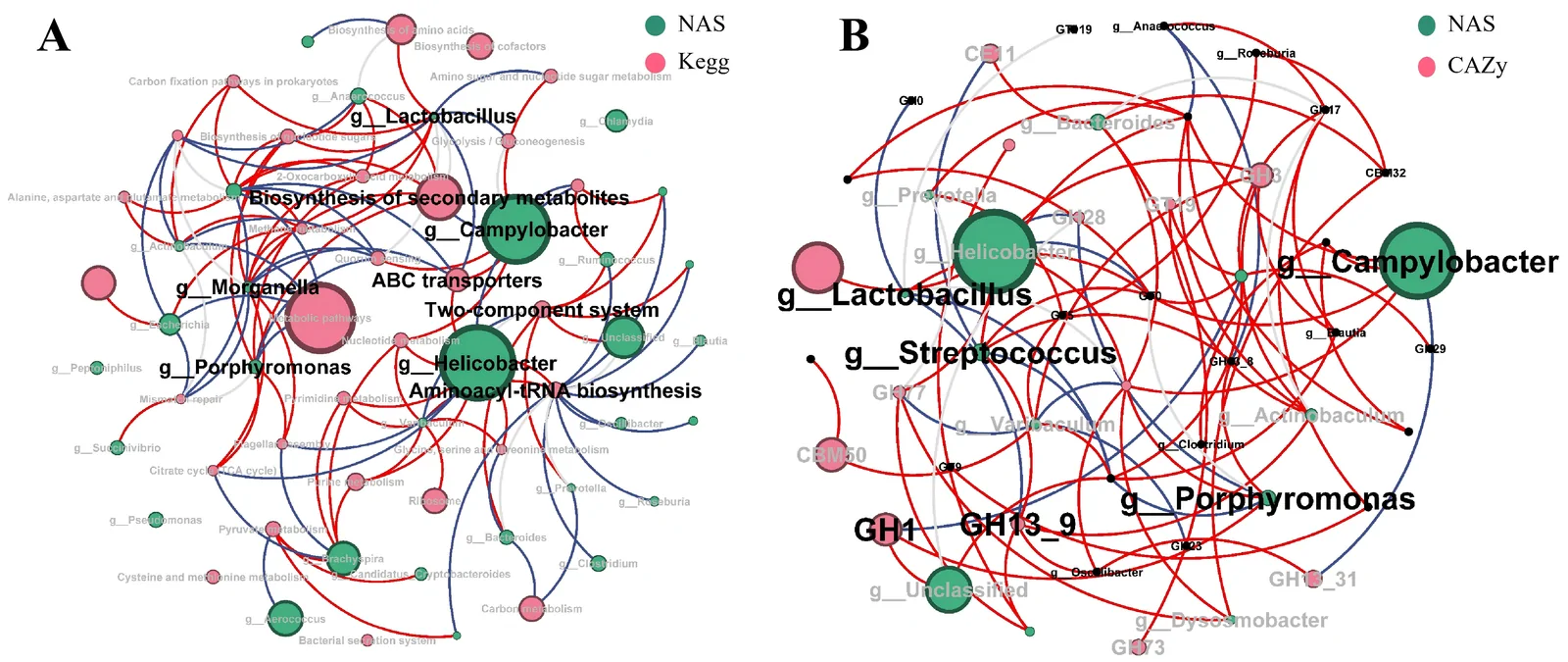
Microbial-KEGG/CAZy co-occurrence networks in INT microbiomes. (**A**): Co-occurrence network of core microbes and KEGG pathways in the top 30 INT microbiome. (**B**): Co-occurrence network of core microbes and CAZy families in the top 30 INT microbiome. Red lines show a positive relationship with r < 0.9, while blue lines show a negative relationship with r > −0.9. Gray lines show a relationship with |r| > 0.9.

Similarly, in the INT genus–KEGG network (Figure 6A), the 104 exploratory edges at 0.6 ≤ |r| < 0.9 were spread across many pathways; the two with the most genus associations were aminoacyl-tRNA biosynthesis and two-component system. Aminoacyl-tRNA biosynthesis was positively associated with *Dysosmobacter* (r = +0.88), *Pseudoflavonifractor* (r = +0.83), and *Ruminococcus* (r = +0.79) but negatively with *Varibaculum* (r = −0.81) and *Helicobacter* (r = −0.76). The two-component system showed negative links with *Bacteroides* (r = −0.88), *Dysosmobacter* (r = −0.86), and *Pseudoflavonifractor* (r = −0.83) but a positive link with *Varibaculum* (r = +0.88). A further set of pathways each carried five exploratory edges, including ABC transporters (*Campylobacter* r = −0.86, *Lactobacillus* r = +0.83), quorum sensing, methane metabolism, metabolic pathways, and biosynthesis of nucleotide sugars. Full edge lists are in Supplementary Table S8-2. In the INT genus–CAZy network (Figure 6B), no edge survived BH–FDR correction (minimum q_BH = 0.078). Among these non-significant associations, the most highly connected genera were *Helicobacter*, *Streptococcus*, *Porphyromonas*, and *Campylobacter*.

## 4. Discussion

The nasopharynx and rectum are critical microbial habitats in mammals, harboring complex microbial communities that play essential roles in host health, nutrient metabolism, and immune regulation [3,28]. Here, we describe spatially specialized microbial and functional profiles in primate microbiomes. These findings advance our understanding of microbial ecology in *Rhinopithecus roxellana* and may inform health surveillance of endangered species.

### 4.1. Spatially Specialized Taxonomic Architecture

The nasopharyngeal (NAS) and rectal (INT) niches assembled into markedly different communities. The NAS microbiota was dominated by Proteobacteria, resembling the upper respiratory tract microbiota of children [29], swine [30], bovines [31], and ovine species [32]. Notably, *Dolosigranulum pigrum* was highly abundant in the NAS (19.43%) and identified as a key biomarker (LEfSe; LDA = 5.04), with a substantially higher abundance than previously reported in human children (about 5%) [29]. Recent research has shown that *D. pigrum* is increasingly recognized as a commensal with competitive-exclusion activity against airway pathogens in humans [33], and higher nasopharyngeal abundance has been linked to reduced pathogen acquisition in infants [34]. Its enrichment in the monkey nasopharynx may therefore reflect a colonization-resistant, health-associated assembly, although this interpretation remains to be tested.

In contrast, the INT microbiome was dominated by Campylobacterota, with *Campylobacter* and specifically *C. troglodytis* as the principal taxa. This composition markedly deviates from the conventional paradigm that primate gut microbiota are primarily dominated by Firmicutes and Bacteroidetes [8,12,35]. Although *Campylobacter* is conventionally regarded as a pathogenic bacterium in the intestines of humans and animals [36], *Campylobacters* are also commensal organisms that live in the gastrointestinal tracts of birds [37]. More importantly, *C. troglodytis* has been detected in asymptomatic wild chimpanzees [38], and other *Campylobacter* species have similarly been isolated from asymptomatic nonhuman primates, including common marmosets, lemurs, and cotton-top tamarins [39], indicating that *Campylobacter* carriage does not inevitably cause disease. We therefore propose a hypothesized commensal carriage model for *C. troglodytis* in this host, rather than a pathobiont model; this remains to be tested.

Together, the divergent NAS and INT profiles indicate that local anatomical niche shapes community assembly in *Rhinopithecus roxellana*, consistent with cross-body-site and cross-segment studies in humans and livestock [40,41].

### 4.2. Niche-Specific Predicted Potential Metabolic Differentiation

To explore whether the marked taxonomic divergence between NAS and INT is accompanied by functional differentiation, we next compared their predicted metabolic potentials based on metagenomic annotations. Metagenomic functional analysis indicated niche-differentiated metabolic potentials. In the nasopharynx (NAS), the annotated gene content was enriched in amino acid catabolic pathways, including BCAA (valine, leucine, isoleucine), glycine/serine/threonine, and tryptophan metabolism—alongside fatty acid degradation and detoxification modules (benzoate, drug metabolism-cytochrome P450). Similar capacities have been reported in other airway microbiomes: genome-resolved metagenomics of cattle nasal mucus recovered MAGs whose functional annotation revealed enrichment in amino acid and short-chain fatty acid metabolism [42], and human nasopharyngeal communities show disease-associated branched-chain amino acid metabolism [43]. In other systems, several of these annotated pathways have been linked to host-relevant processes: BCAA catabolism can interface with energy production and mTORC1 signaling [44,45]; tryptophan metabolism has been implicated in neuro-immune cross-talk in other models [46], and fatty acid degradation maintains energy homeostasis [47,48]. We emphasize that these interpretations are based solely on genomic predictions. Whether the NAS-enriched BCAA, tryptophan, and fatty acid modules actually engage these host pathways in *Rhinopithecus roxellana* remains untested and will require targeted metabolomic and transcriptomic validation.

By contrast, the INT microbiome was enriched in ribosome-associated pathways, amino sugar and nucleotide sugar metabolism (providing precursors for bacterial cell-wall and capsule synthesis) [49], methane metabolism (consistent with the established role of hydrogenotrophic methanogenesis in maintaining gut hydrogen homeostasis) [50,51], and the biosynthesis of various antibiotics and other secondary metabolites [52]. These patterns are consistent with prior characterizations of the gut microbiome in golden snub-nosed monkeys [7,12,35]. These pathway-level annotations describe which metabolic transformations are theoretically possible, but they do not by themselves resolve the enzymatic machinery or host interaction capacity that underpins niche colonization. To sharpen the functional picture beyond KEGG-level pathways, we therefore examined two complementary gene categories: the carbohydrate-active enzyme (CAZy) repertoire and the pathogen–host interaction (PHI) gene set.

Twenty-nine differentially abundant CAZy families were nasopharyngeal—including glycoside hydrolases (GH13_29, GH39, GH103) and a sialate O-acetylesterase (CE14) acting on host sialic-acid glycans—pointing to an airway toolkit for host-glycan foraging rather than gut-type plant-fiber degradation [53,54,55]. PHI profiling was even more asymmetric: of 134 categories, 126 were NAS-enriched against only eight intestinal, and the few INT-restricted functions—exemplified by PHI:7197—mark niche-specific gut colonization strategies [56]. We interpret the NAS bias toward PHI-factor homologs as a signature of an environmentally exposed mucosal niche where commensals maintain adhesion and immune-modulation capacities [57], not as evidence of pathobiology; as with all annotations here, these reflect inferred genetic potential rather than measured activity.

### 4.3. Hub Taxa and Microbial Correlation Networks (Exploratory)

As noted for the within-niche (§3.3) and genus–function (§3.5) networks, the small sample size (n = 8 per niche) means the strongest correlations are the most likely to be spurious. In our networks the largest edges reached |r| ≥ 0.9, and correlations of this magnitude are the classic signature of numerical artifacts arising from compositional structure and low statistical power rather than genuine ecological coupling [58,59]. No edge remained significant after Benjamini–Hochberg (FDR) correction (all qBH > 0.05); therefore, all patterns should be treated as exploratory and hypothesis-generating, not as evidence of ecological interaction.

In the genus-level network interactions, *Treponema* formed a tightly associated module with *Ruminococcus* (r = 0.88). This is biologically plausible because *Treponema* associates with plant fiber and interacts synergistically with cellulolytic bacteria such as *Ruminococcus* to accelerate polysaccharide breakdown [60]. *Treponema* is likewise enriched in nonhuman primates—e.g., it is abundant in the gut of rhesus macaques [61] and shows a lifestyle-associated distribution across chimpanzees, gorillas, and humans [62], consistent with the fiber-related microbial assembly inferred here, although direct *Treponema*–*Ruminococcus* syntrophy has, to our knowledge, been demonstrated chiefly in ruminants [60]. It echoes the *treponeme*-rich, plant-polysaccharide-fermenting gut microbiomes of high-fiber forager populations [63,64]. Though non-significant at n = 8, its direction is biologically plausible. The niche-dependent *Porphyromonas* associations were positive with *Escherichia-Shigella* in the INT but negative with *Stenotrophomonas* in the nasopharynx, which likewise echoes the emerging view that the same genus can occupy distinct interaction contexts across body sites [65,66].

In the genus–KEGG/CAZy correlation networks, *Corynebacterium* and *Dolosigranulum* emerged as the most connected hubs in the NAS functional network, mirroring the well-documented, health-associated *Dolosigranulum*–*Corynebacterium* signature of the upper-airway microbiota in infants and children [33,34]. The exploratory genus–KEGG links most often involved biosynthesis of cofactors, glycolysis, pyrimidine metabolism, and amino acid biosynthesis together with glycine, serine, and threonine metabolism. These pathways are compatible with mucin foraging at the mucosal surface: the degradation of host-secreted mucin glycans, a recognized carbon and energy source for mucosal commensals [67], could release sugars and sulphate that feed the central-energy, nucleotide, and cofactor pathways identified here.

Taken together, the patterns above are literature-consistent but statistically unsupported at n = 8; they frame clear, testable hypotheses for larger, independently sampled cohorts. Confirming these hypotheses will require metatranscriptomic or metabolomic validation of mucin foraging in the nasopharynx and of the *Treponema*–*Ruminococcus* (fiber-degrading) association/module in the rectum, together with strain-resolved tracking of *Porphyromonas* ecotypes across the two sites.

### 4.4. Zoonotic Potential and Implications for Health Surveillance

Metagenomic profiling extends beyond traditional community ecology, offering a non-invasive, culture-independent approach to monitoring the health of this endangered host. By resolving taxonomic signals from fecal and nasopharyngeal samples, the method can highlight candidate taxa for later confirmatory studies; however, read-level detection alone does not indicate active infection or established reservoir status. In the current dataset, the NAS microbiome contained potentially zoonotic or opportunistic taxa, such as *Helicobacter pylori* [68] and *Chlamydia psittaci* [69]. Notably, *H. pylori* has known natural reservoirs in macaques and great apes [68,70], while other zoonotic gastric and enterohepatic Helicobacter species have been documented in great apes [71], whereas *C. psittaci*, which is primarily avian, has been documented in diverse mammals [69,72,73], yet its detection in free-ranging golden snub-nosed monkeys remains rare. Thus, our sequence-based finding requires confirmation via targeted diagnostics (qPCR, culture) before any inference about active carriage or zoonotic risk. Meanwhile, *Campylobacter* was detected in the intestinal microbiome; as a genus with well-known zoonotic risk and documented occurrence in nonhuman primates [74,75], its detection in this endangered host warrants targeted surveillance. The sample size was relatively small (n = 8), and the obtained abundance data cannot be directly extrapolated to population-level prevalence. Under the “One Health” framework, this work still holds significant value: it not only serves the conservation biology of endangered primates, but also can provide early warning for new emerging zoonotic diseases at the human–wildlife interface [76,77,78].

### 4.5. Limitations

This study provides initial insights into the spatial distribution and functional associations of the nasopharyngeal and rectal microbiota in the golden snub-nosed monkey. However, several limitations should be noted. First, the small sample size (n = 8) limits statistical power and the stability of network analyses; some SparCC coefficients approached extreme values and were treated as artifacts. Second, the lack of temporal data precludes analysis of seasonal dynamics. Third, metagenomic functional prediction cannot distinguish actively expressed genes [79] or capture metabolite profiles [80]; multi-omics integration (e.g., metatranscriptomics, metabolomics) is needed [81]. Fourth, taxonomic and pathogen assignments used permissive thresholds (identity ≥60%, coverage ≥15%) and no extraction/sequencing negative controls or independent molecular confirmation were included; thus, pathogen-associated observations are descriptive and hypothesis-generating rather than confirmed infections, and two NAS samples had low post-host-removal read depth that reduces confidence in species-level calls. Fifth, the ecological and pathogenic significance of dominant taxa such as *Campylobacter* remains unclear in this endangered host.

## 5. Conclusions

This study reveals niche-specific microbial architectures in the nasopharynx and rectum of golden snub-nosed monkeys. The two sites differ taxonomically and in their predicted functional potential, with the NAS oriented toward catabolic/detoxification pathways and the INT toward ribosome, sugar metabolism, and biosynthesis pathways. Differentially abundant and highly connected taxa (e.g., *Dolosigranulum* in NAS, *Campylobacter* in INT) are hypothesized to contribute to spatially specialized community assembly, but these roles remain to be validated. These findings provide a foundational microbiome atlas for this endangered primate and highlight the value of multi-niche perspectives in conservation health monitoring; larger, longitudinal, and technically replicated cohorts are required to test the hypotheses raised here.

## Data Availability

Full-length 16 s rRNA gene sequencing raw reads were deposited in the National Center for Biotechnology Information (NCBI) Sequence Read Archive (SRA) database (Accession Number: PRJNA1308553). Metagenomic genome sequencing data was submitted to the China National Center for Bioinformatics (CNCB), under BioProject accession number PRJCA044952.

## Supplementary Materials

The following supporting information can be downloaded at: https://www.mdpi.com/article/10.3390/biology15171530/s1.
