# Supplementary material for: Spatially Specialized Nasopharynx–Rectal Microbiomes in Golden Snub-Nosed Monkeys (Rhinopithecus roxellana)
Source: Biology (Basel). 2026 Sep 3;15(17):1530. doi: 10.3390/biology15171530 (PMC13564790; doi:10.3390/biology15171530)
Supplement: Supplementary file 1 [file biology-15-01530-s001.zip › Supplementary Figures S1-S3.pdf]

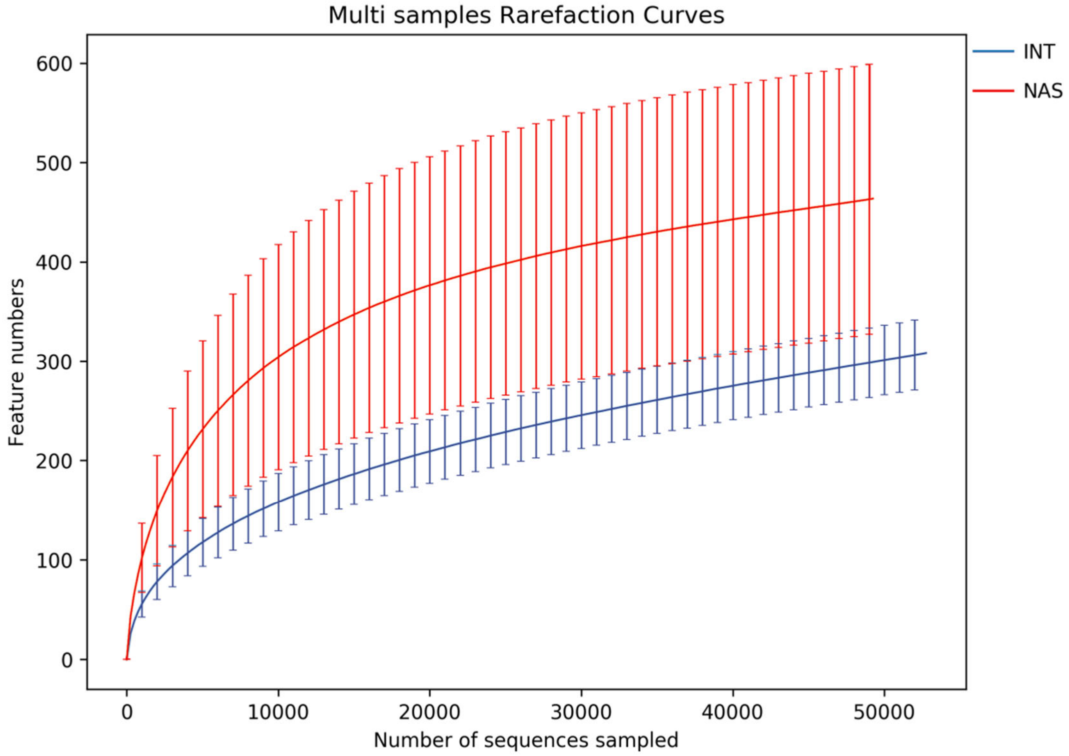

Supplementary Figure S1. Rarefaction.curve_group.

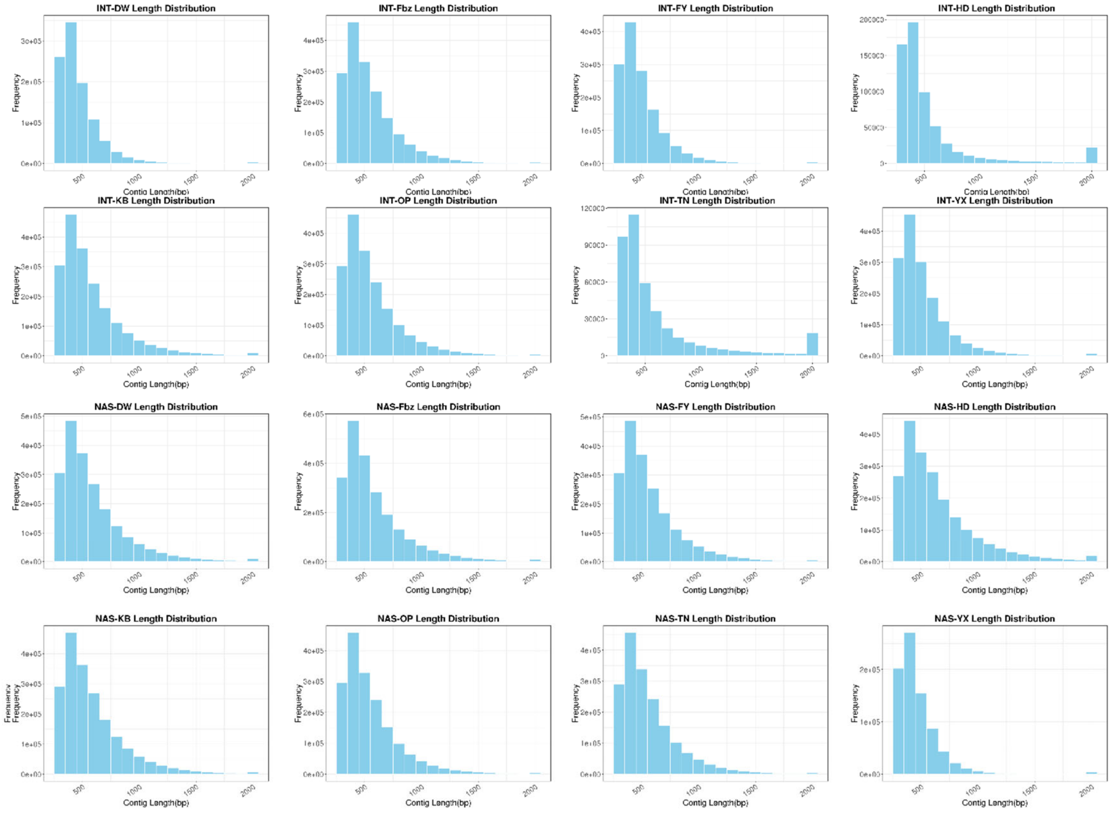

Supplementary Figure S2. Read length distribution characteristics across different experimental sample groups.

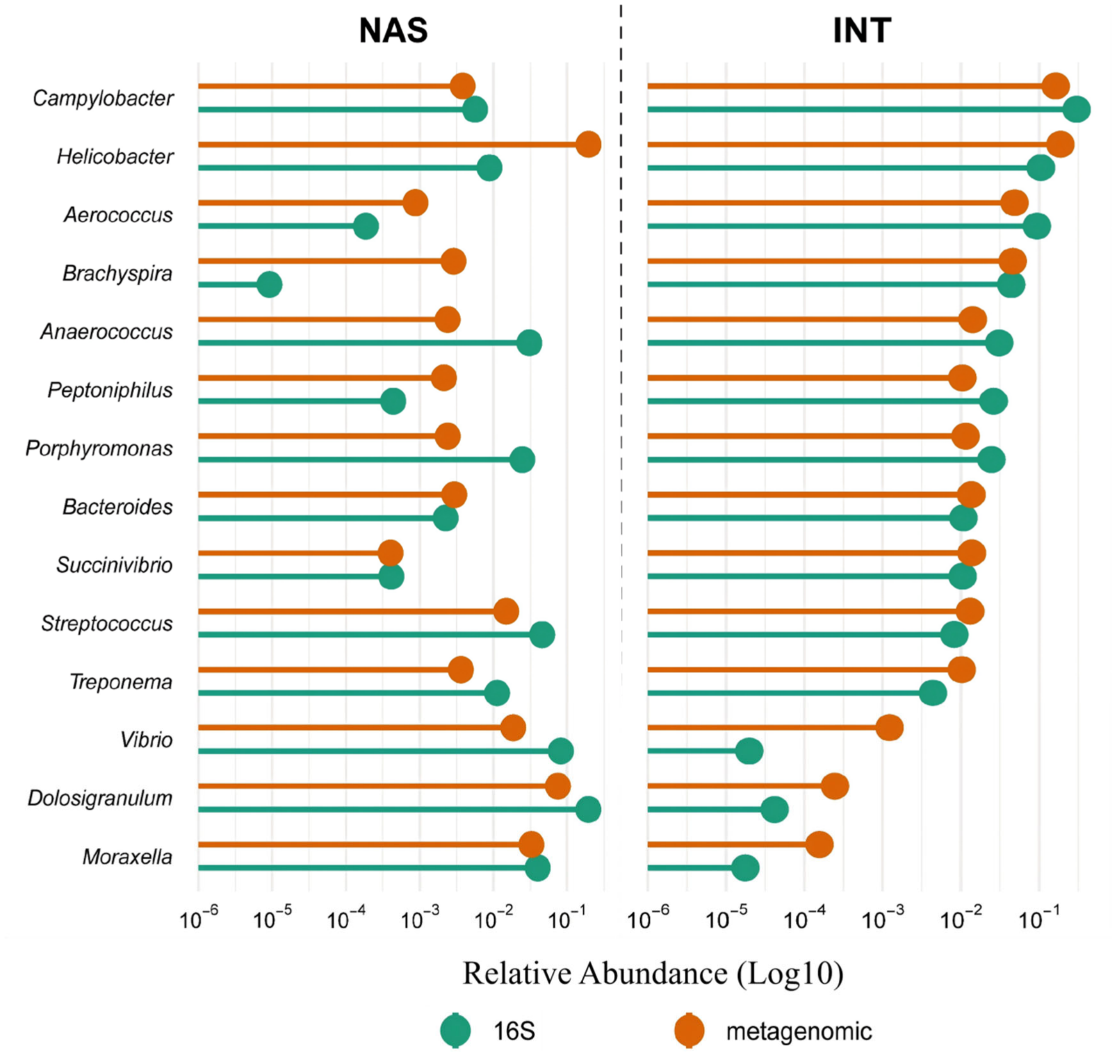

Supplementary Figure S3. Genus abundance profiles of INT and NAS microbiota detected by metagenomics and 16S rRNA sequencing.
